# 

**Published:** 1877-11

**Authors:** 


### CONTENTS:

ARTICLE I.— The Action of the Nervous System. By John J. Caldwell, M. D..... 289

ARTICLE II.— The Development of Dentine. By C. Sihler, M. D........ ......... 298

ARTICLE III— Should Five Years' Practice in Dentistry, Inclusive of Pupilage, Continue to be Regarded as Equivalent to One Course of Lectures inDental Colleges. By John H McQuillen, M D., D. D. S........ 314

ARTICLE IV.— The Limits of the Optical Capacity of the Microscope. By Lester Curtis, M. D........................................... 322

### EDITORIAL, ETC.

The Decline of Mechanical Dentistry.—Its Appropriateness.................. 328
Letter Concerning the Southern Dental Association.. .......................... 330
The Present Number of "JOURNAL"........................................ 331
Harris' Medical and Dental Dictionary.—A New Edition...................... 331

### NOTES FROM PRACTICE.

Practical Hints in Dental Mechanism........................................ 332

Broaches for Pulp Canals .......... ........................................ 332

A Case in Practice.................................. ....................... 333

Anæsthetic Effects of Cold Water............................................ 333

### MONTHLY SUMMARY.

Advertising Dentists.................................................... 333

Decline of Mechanical Dentistry............................................ 334

Davyum......................................................... 334

Diverse and Divers Views.................................................. 335

Eucalyptus as a Local Anæsthetic ........................................ 335

Strychnia as a Substitute for Quinia......................................... 335

Malaria ............ . ........................................................ 336

Secondary Dentine........................................................ 336

Animal Magnetism........................................................ 336

